# Endoscopic observations as a tool to define underlying pathology in kidney stone formers

**DOI:** 10.1007/s00345-018-02616-3

**Published:** 2019-01-04

**Authors:** Maria Sloth Pless, James Caldwell Williams, Kim Hovgaard Andreassen, Helene Ulrich Jung, Susanne Sloth Osther, Dorte Ravnsmed Christensen, Palle Jörn Sloth Osther

**Affiliations:** 1 Urological Research Center (URC), Department of Regional Health Research, University of Southern Denmark, Vejle, Denmark; 2 Department of Anatomy and Cell Biology, Indiana University School of Medicine, Indianapolis, IN USA; 3 Department of Urology, Lillebaelt Hospital, Beriderbakken 4, Vejle, Denmark

**Keywords:** Kidney calculi, Papillae, Ureterorenoscopy, Randall’s plaque, Pathology, Micro-computerized tomography

## Abstract

**Purpose:**

Advancements in endoscopy offer the possibility of inspection of intrarenal anatomy and pathology. The aim of the study was to evaluate renal papillary appearance in kidney stone formers and to correlate papillary findings with stone type and patient metabolic data.

**Materials and methods:**

A consecutive cohort of 46 kidney stone formers undergoing retrograde intrarenal surgery was enrolled. During surgery, renal papillae were characterized in the domains of ductal Plugging (DP), surface Pitting, Loss of papillary contour, and Amount of Randall’s plaque (RP, PPLA scoring). Stone material was analyzed using micro-CT and infrared spectroscopy, and blood and urine were collected for metabolic evaluation.

**Results:**

In all patients, renal papillae had changes in at least one of the domains of the PPLA score. Examining the total population, it was evident that patients with predominantly plugging (DP > 0) all had very low RP scores. There were no significant trends between mean PPLA scores and urinary analytes for the total group.

**Conclusion:**

Efforts to prevent renal stone formation have so far been insufficient in majority of patients. Digital endoscopy reveals that kidney stone formers have different and distinct papillary morphologies that seem to be linked to specific stone-forming pathways. Since renal papillary abnormalities may be easily identified during endoscopy, this may in the future prove to be an important method for tailoring prevention strategies in kidney stone patients.

## Introduction

Nephrolithiasis is a common disease throughout the world; the lifetime risk of developing kidney stones in Europe and USA is estimated to be 10–15% [1, 2] and kidney stone disease represents a major burden to quality of life [3].

Eight decades ago, Alexander Randall proposed sub-epithelial calcium phosphate deposits at the tip of the renal papillae as the origin of renal calculi [4]. His findings were not adequately recognized as an important step forward in our understanding of the pathogenesis of renal stone formation, until recent research using modern investigational tools brought the unique findings into further perspective [5, 6]. It is now widely accepted that some kidney stones develop attached to sub-epithelial plaques of calcium phosphate crystals (Randall’s plaques) [7]. Other stones form as a result of occlusion of the openings of the ducts of Bellini by stone-forming crystals (ductal plugs) [5]. These plaques and plugs eventually extrude into the urinary space, acting as nidi for crystal overgrowth (i.e., calcium oxalate, CaO_*x*_) and stone formation [8]. Randall’s plaques begin as deposits of apatite in the basement membranes of the thin limbs of Henle’s loop and can grow to become extensive deposits in the interstitial space beneath the epithelium covering the papillary surface [5, 9]. The fraction of the papilla surface that is covered with Randall’s plaque correlates with number of stone episodes and calcium excretion in the individual stone patient [10, 11]. These findings seem to be unique for those patients forming stones on RP, whereas other histopathological findings with intratubular crystals have been identified as unique for stone formers with other types of stone diseases [12, 13]. The exact mechanisms by which these histopathological and ultrastructural changes occur remain unclear.

Technologic advancements in digital endoscopy offer the possibility of detailed inspection of intrarenal anatomy and pathology [14]. Recently, attention has been drawn to the relation between distinct renal papillary abnormalities visualized during endoscopy and unique pathogenetic pathways of stone formation [15–17]. The aim of the present study was to evaluate renal papillary appearance in a consecutive cohort of kidney stone formers undergoing retrograde intrarenal stone surgery (RIRS) using digital flexible ureteroscopes and to correlate the papillary findings with stone type and patient metabolic data to explore the role of endoscopic papillary findings in defining underlying stone-forming mechanisms and clinical outcomes.

## Methods

### Ethics

The study was approved by the Scientific Ethical Committee of the Region of Southern Denmark (ID: S-20162000-2,) and the Danish Data Protection Agency. Apart from visual mapping of the renal papillae, the endoscopic procedure for stone management (RIRS) did not differ from standard surgical routine. Therefore, informed consent was waived.

### Patients

In the period February to July 2016, patients aged 18 years or above with renal stones admitted for RIRS at Lillebaelt Hospital, Denmark were consecutively included in the study. Characteristics of the patients are presented in Table 1. Inclusion criteria consisted of standard indications for performing RIRS. Thus, included stone patients were not selected according to the suspicion of specific underlying stone diseases and, thus, represented an unselected cohort of kidney stone formers.

**Table 1:** Characteristics of patients included

Clinical characteristics	No patients [*n* = 46 (range)]
Age	54 (20–84)
BMI	27.5 (18.6–47.9)
Male	20
Female	26
Years with stone disease	6.4 (0–50)

Values are means (ranges) and absolute numbers (gender)

*BMI* body mass index

### Blood and urine samples

All patients routinely provided 24-h urines (24-h) on unrestricted diet before surgery or a minimum of 8 weeks later. No patients were on medications for stone prevention. On the day of surgery, a routine blood sample was drawn and fasting morning spot urine was collected for immediate pH measurement. Blood samples were analyzed for plasma creatinine, uric acid, sodium, potassium, ionized calcium, bicarbonate and phosphate. The 24-h urine sample was analyzed for volume, citrate, oxalate, calcium and creatinine using standard laboratory methods.

### Stone analysis

Stones collected during surgery were routinely analyzed using Fourier-transform infrared spectroscopy (IR) at the laboratory of Aalborg University Hospital, Denmark. Additional stone samples were obtained from most patients and were analyzed using both IR and micro-CT to obtain stone morphological details. Micro-CT was performed on specimens in vitro, using a Skyscan 1172 system [18]. Scans utilized 60 kV with a 0.5 mm Al filter, rotating the specimen 0.7° for each X-ray image. Reconstruction voxel size was 4.8 μm for images shown.

### Endoscopic grading of renal papillary appearance

**Fig. 1:**
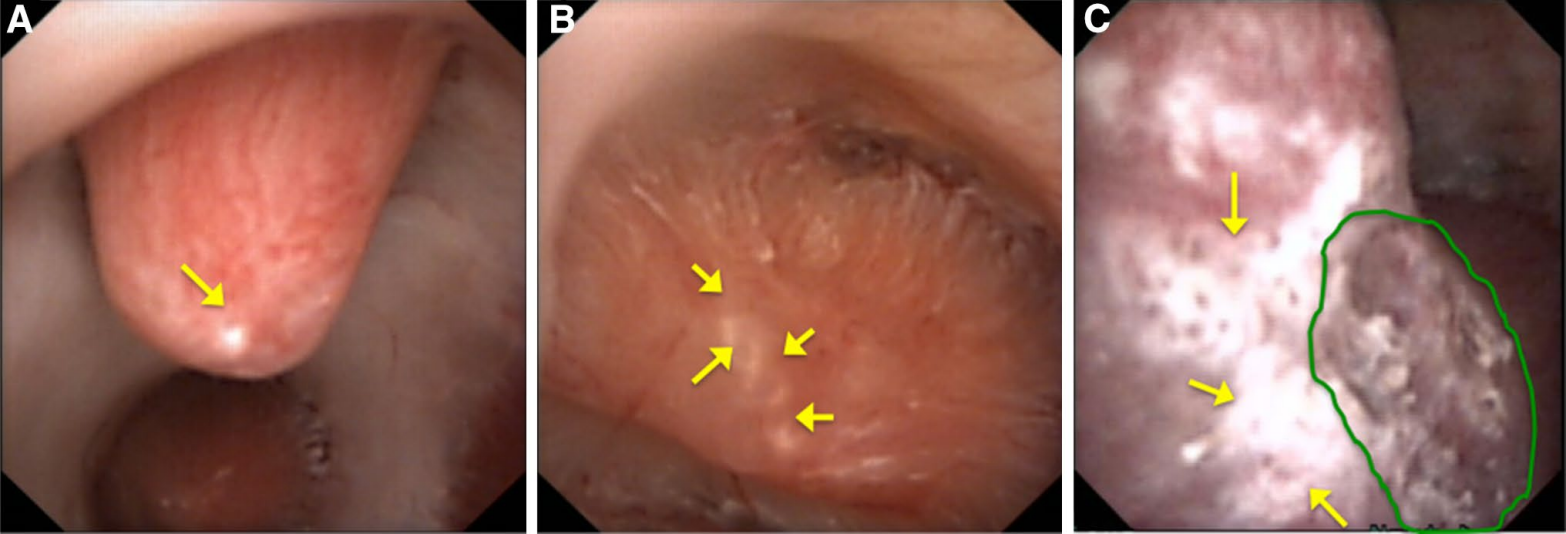
Examples of grades of Randall’s plaque and pitting in the PPLA (plugging, pitting, loss of contour, are of RP) system. **a** Mild Randall’s plaque on a perfectly normal papilla (score of 0, 0, 0, 0), **b** Moderate Randall’s plaque (RP score of 1) with a few linear white lines that are thought to be ductal plugs (score of 1, 0, 0, 1), **c** Severe Randall’s plaque (score of 2) which also shows surface pitting (surrounded by a green line) with no plugging and normal contour (score of 0, 2, 0, 2). This kind of shallow surface pitting is thought to be a result of previous loss of stones that had grown on Randall’s plaque and then released [xx]

**Fig. 2:**
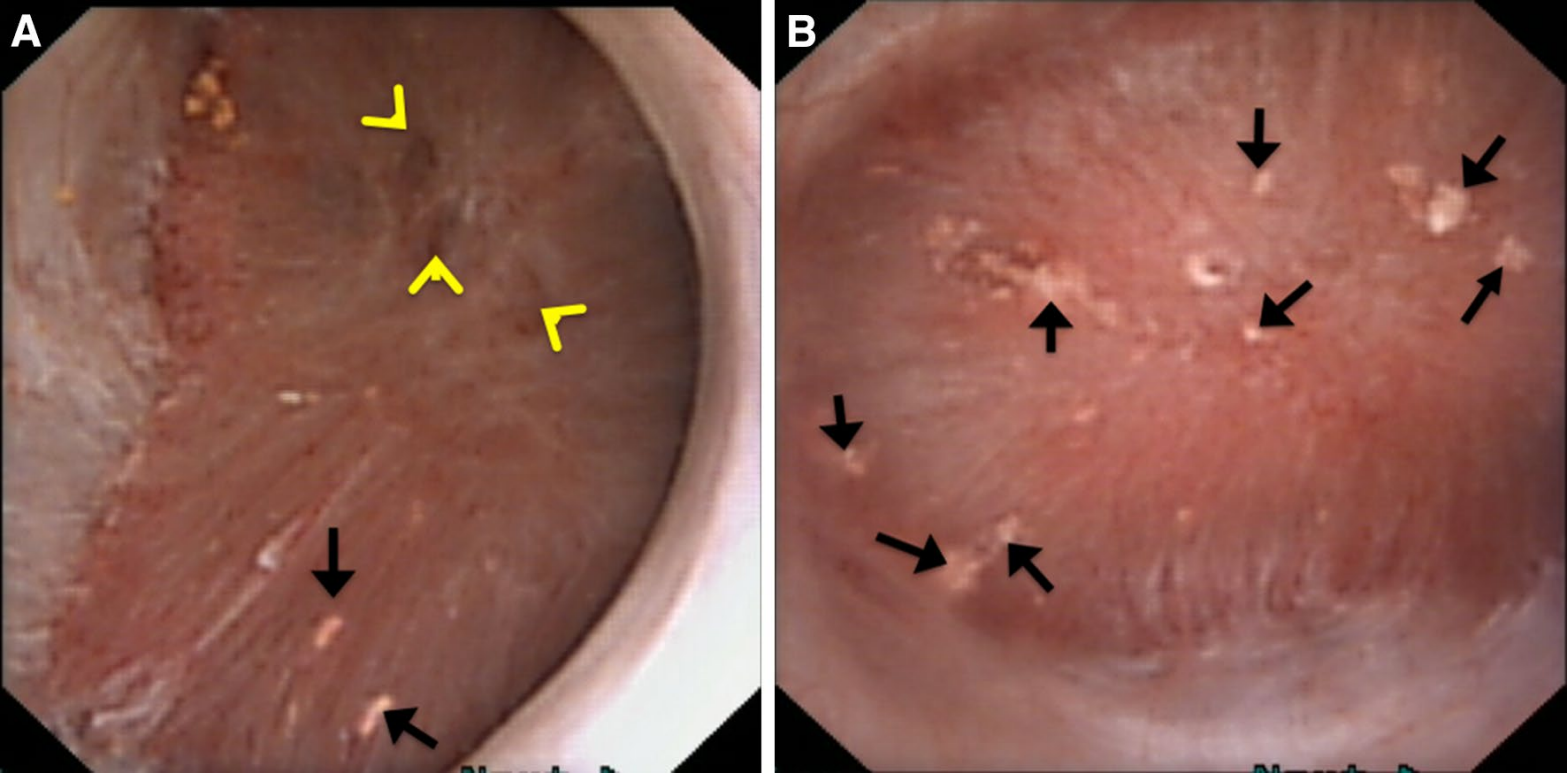
Examples of grades of ductal plugging and loss of contour in the PPLA system. **a** Yellow arrowheads show dilated ducts and black arrows show ductal plugs. The papilla is flattened and illustrates loss of contour. **b** Black arrows show ductal plugs

The PPLA scoring system (Table 2) was used for endoscopic grading of renal papillary findings. Details on the scoring system have recently been published by Borofsky et al. [14]. The PPLA system—grading papillary appearance in the domains of ductal plugging (DP), surface pitting (SP), loss of contour (LC) and Randall’s plaque (RP)—was designed to simplify the description of papillae during endoscopy as a clinical and research tool to explore the significance of papillary pathology in stone formation. The different papillary pathologies are presented in Figs. 1 and 2 and further detailed in Table 2. All lesions except RP are thought to represent different degrees of nephron loss [14]. In the present study, the original PPLA grading score was modified using numerical values for RP scoring in line with the other domains as suggested by Cohen et al. [17]. Each papilla was scored in all domains according to Table 2 and a final PPLA score for each papilla was calculated. After identifying and assigning scores to all accessible papillae within the renal unit, a mean PPLA score was calculated by dividing the sum of papilla scores by the number of papillae examined. In addition, the lowest and the highest scores are presented to denote range of pathology encountered. Patients with medullary sponge kidney (MSK) were considered separately, as they are recognized as having a unique stone pathogenesis unlike other common stone-forming conditions [19].

**Table 2:** PPLA scoring system for grading renal papillae in kidney stone formers

Score	0	1	2
Ductal plugging^a^	0 yellow plaque deposits/dilated ducts	≤ 5 yellow plaque deposits/dilated ducts	> 5 yellow deposits/dilated ducts
Surface pitting^b^	None	≤ 25% papillary surface involved	> 25% papillary surface involved
Loss of contour^c^	None	Depressed	Completely flattened
Amount of Randall’s plaque^d^	Mild	Moderate	Severe

^a^Plugging presents as yellow intraductal mineral deposits visualized just under the urothelial surface or protruding from the mouth of the dilated duct itself (Fig. 2). An empty dilated duct is also indicative of plugging (Fig. 2)

^b^Pitting shows as a crater-like focal erosion of the papillary surface that most likely represents a mechanical disruption due to a detached stone (Fig. 1)

^c^Loss of contour is characterized as progressive flattening of the papilla, and represents an advanced stage of papillary injury (Fig. 2)

^d^Randall’s plaques are characteristically white most commonly located near the tip of the papilla but with potential to appear anywhere on its surface (Fig. 1)

Two kinds of digital ureterorenoscopes were used: Storz Flex XC (Karl Storz Endoskope, Tütlingen, Germany) and Olympus URF-V2 (Olympus, Hamburg, Germany). The location of each papilla was determined using fluoroscopy with contrast instillation through the ureteroscope. The papillae were systematically evaluated and videotaped during flexible ureteroscopy after stone material had been removed.

### Image analysis

Representative still images of each papilla were captured from the video footage. The first author (MSP) initially graded the papillae during all surgical procedures. MSP and JCW reviewed the videos and images to determine location and appearance of papillae for final PPLA scoring.

### Statistics

Statistical analyses were performed using JMP (SAS^®^, Inc. Cary, NC, USA) and STATA 14 (STATA^®^ Corp., College Station TX, USA). Bivariate associations of PPLA scores and subscores with blood and urine parameters were initially evaluated using Spearman’s Rank Correlation and the Rank sum test. One-way analysis of variance (ANOVA) was used to determine whether there were any differences between urinary parameters and PPLA scores. All reported *p* values were two sided, with *p* < 0.05 considered statistically significant.

## Results

### Patients and stone classification

This unselected, consecutive series include 46 patients, of which 20 were males and 26 females (Table 1). Based on IR and micro-CT, patients were classified as calcium oxalate (CaO_*x*_) stone formers if their stones contained > 50% CaO_*x*_ monohydrate (COM), CaO_*x*_ dihydrate (COD) or a combination of COM/COD. Patients with > 50% apatite in their stones were classified as apatite stone formers. Patients with stones containing any struvite were classified as such and patients classified as uric acid stone formers had stones containing > 50% uric acid [20]. Thus, 25 patients (54%) were classified as CaO_*x*_ stone formers, two of which previously had undergone bariatric surgery; 5 were struvite stone formers (11%); 5 (11%) were uric acid stone formers; and 5 (11%) apatite stone formers. In 4 patients, stone fragments were not available for analysis (9%). Based on radiologic and endoscopic findings, two patients (4%) had medullary sponge kidney (MSK) and these patients formed their own group.

### Endoscopic papillary findings

In all patients, the renal papillae had changes in at least one of the domains of the PPLA system. In CaO_*x*_ stone formers, both RP (12 patients (48%)) and ductal plugs (DP) [9 patients (36%)] were seen with mean RP scores ranging from 0.13 to 1.43 and mean DP scores ranging from 0.13 to 0.63; thus, RP was predominant in this group. In 20 of 25 CaO_*x*_ stone formers (80%), the renal papillae were seen as having some loss of papillary volume with LC mean score ranging from 0.13 to 2.0. Pitting of the papillae was seen in 6 CaO_*x*_ patients with mean SP scores ranging from 0.10 to 1.38.

The apatite group was predominantly characterized by DP [3 patients (60%)] and LC (4 patients (80%)), with mean DP and LC scores ranging from 0.38 to 0.57 and 0.63 to 2.0, respectively. SP was not seen in the apatite group and only one patient showed any Randall’s plaque (mean patient RP score 0.44). One patient in this group had slightly elevated levels of plasma parathyroid hormone (7.9 picomol/l) and also showed the highest mean DP score of 0.57.

Uric acid stone formers were characterized by both RP (2 patients (40%)) and DP [3 patients (60%)) with mean RP scores ranging from 0.38 to 1.14, and mean DP scores from 0.38 to 0.50)]. Four uric acid patients (80%) showed LC and 2 patients (40%) showed SP with mean LC and SP scores from 0.63 to 2.00 and 0.07 to 0.63, respectively.

In struvite stone formers, RP was not seen and DP was seen in only one of the four patients (20%). However, LC was marked in the majority of patients (4 of 5 patients (80%)) with mean LC scores ranging from 1.33 to 2.00.

The 2 MSK patients had distinctive and widespread papillary changes, including mild RP, extensive DP, and LC, confirming that these patients differ from other stone formers with regard to pathology of stone formation [19].

In the total population of 46 stone formers, LC was identified in 38 patients (83%) with a total mean LC score of 1.20 ranging from 0.13 to 2.00.

**Fig. 3:**
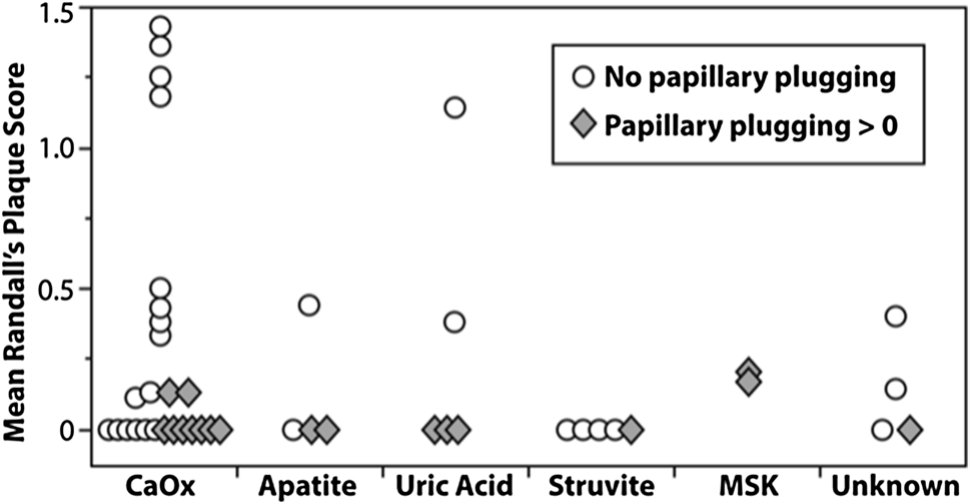
Relation between visible mineral plugging of papillary ducts and Randall’s plaque in subgroups of renal stone formers. *CaO*_*x*_ calcium oxalate, *MSK* medullary sponge kidney

**Fig. 4:**
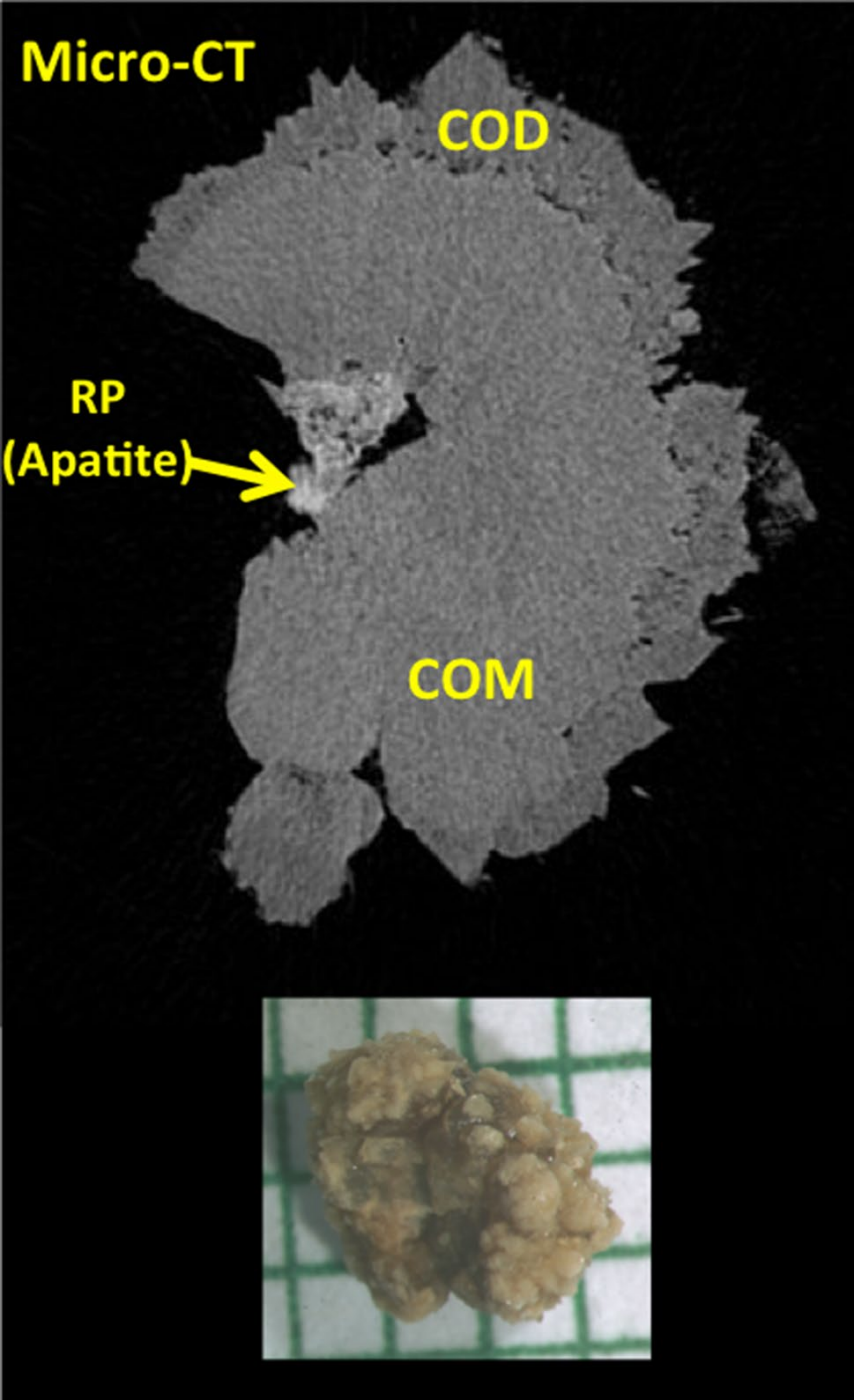
Micro-CT image slice from a stone that grew on Randall’s plaque, with photo of stone on mm paper. A 58-year-old male with BMI 21.7 from the CaO_*x*_ group had three stones analyzed with micro-CT and IR; two of these showed Randall’s plaque (RP) attachment sites (apatite) in the stones (micro-CT), one shown here. Patient’s mean RP score was rather low (0.25). However, papillary endoscopic images showed attachment of stones to Randall’s plaque (Fig. 5), consistent with findings by micro-CT that stones in this patient developed as overgrowth on Randall’s plaque. In this patient occurrence of Randall’s plaques was accompanied by mild hypercalciuria (8.6 mmol/24-h) and low urine pH (5.5). COM, calcium oxalate monohydrate; COD, calcium oxalate monohydrate

**Fig. 5:**
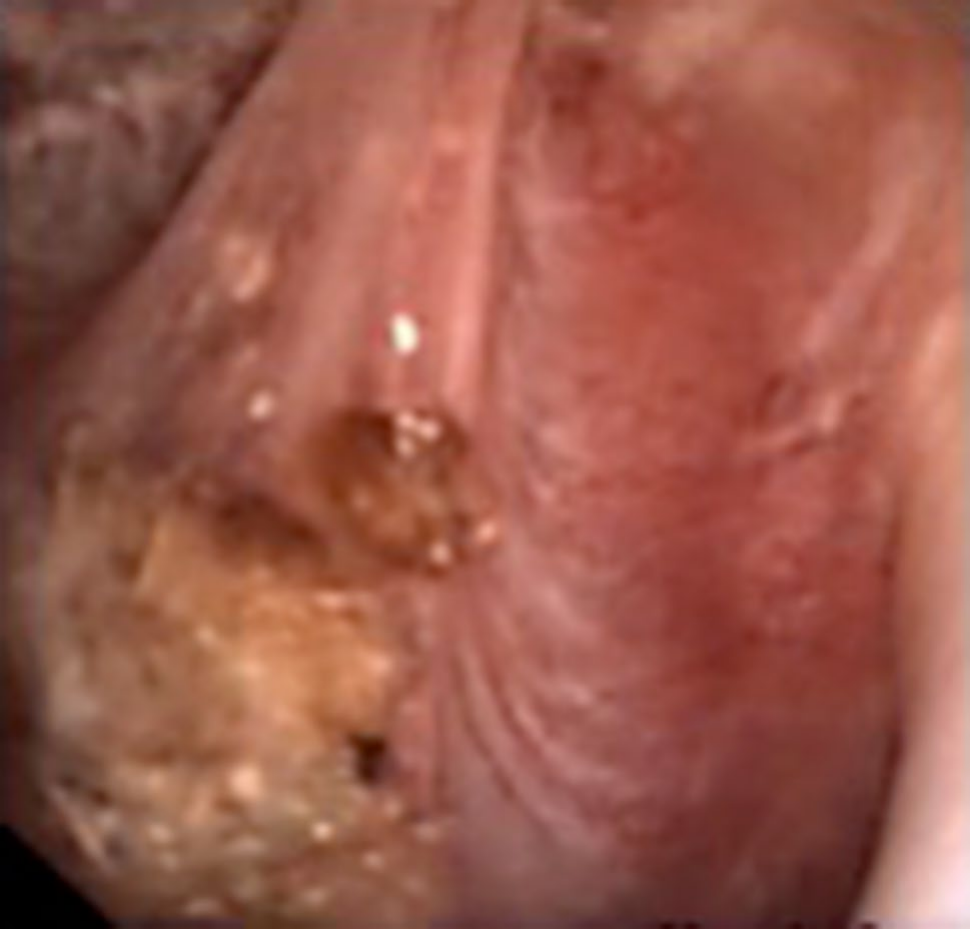
Endoscopic view of stones attached to the renal papilla in the same patient as Fig. 4

**Fig. 6:**
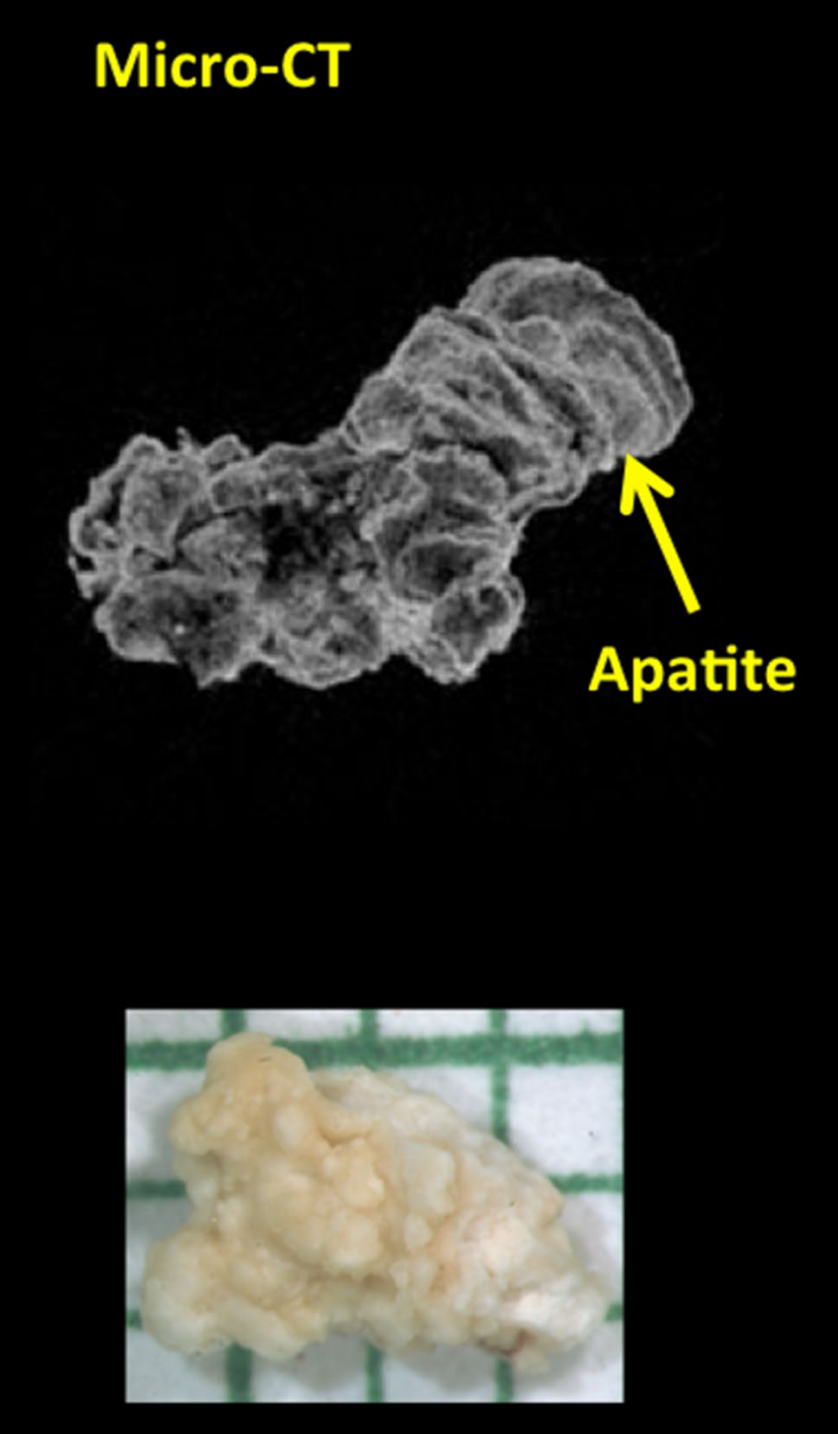
Apatite stone former with hyperparathyroidism. A 45-year-old male with BMI 36.1 had 3 stone specimens analyzed using IR and micro-CT, both showing apatite. 24-h urine revealed a very high urinary calcium excretion rate (17 mmol/24-h) and high urine pH (6.5). This patient was diagnosed with primary hyperparathyroidism with plasma-ionized calcium of 1.69 mmol/l and plasmaparathyroid hormone (P-PTH) of 7.9 picomol/l. Papillary findings were characterized by ductal plugging (DP) and loss of contour (LC), with mean DP and LC scores of 0.57 and 1.0, respectively. No Randall’s plaque and surface pitting were seen in this patient

Examining the total patient series, it was evident that patients with predominantly plugging (DP > 0) all had very low RP scores (Fig. 3). Two illustrative cases of RP and DP stone disease, respectively, are presented in Figs. 4, 5 and 6.

### Compound papilla findings

**Fig. 7:**
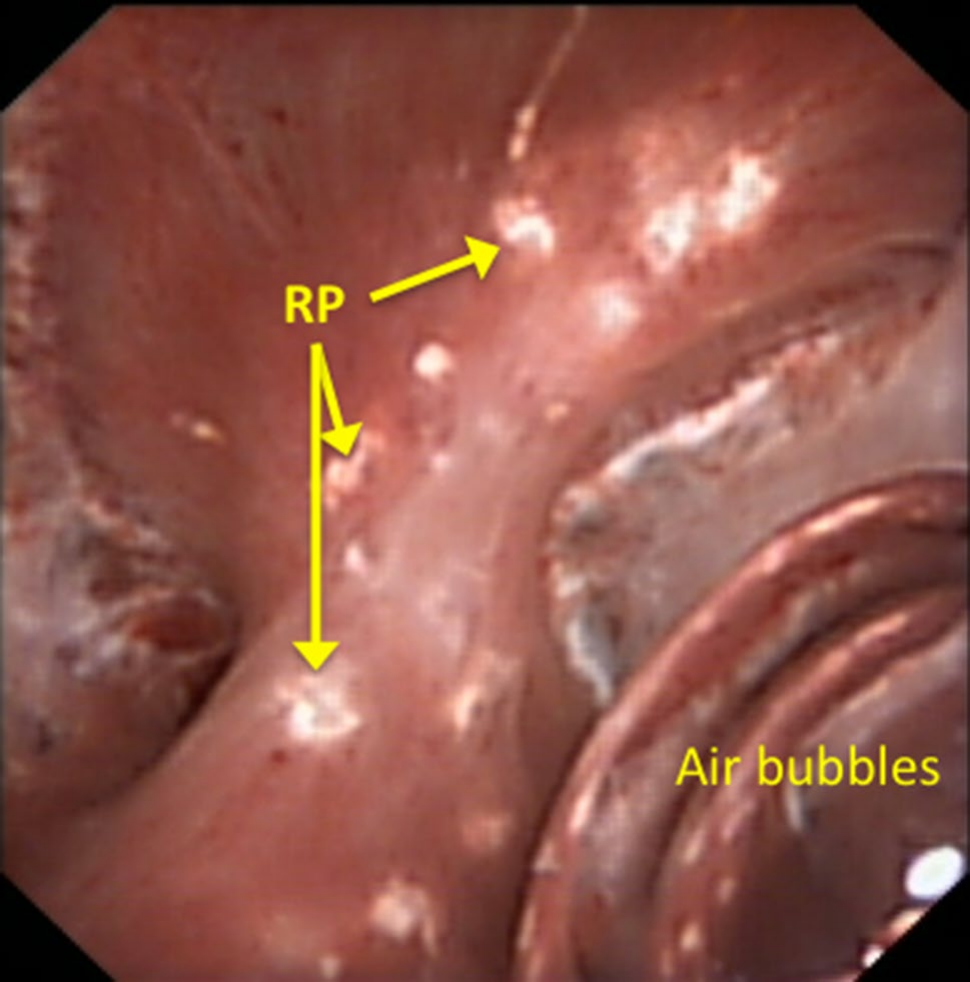
Compound papilla showing areas of Randall’s plaque (RP)

Compound papillae were not infrequent in this patient cohort (Fig. 7). On average, there were 7.6 ± 2.3 accessible papillae per patient kidney, with 24.1 ± 23.9% of the papillae being compound. 67% of the kidneys had at least 1 compound papilla.

### Blood parameters

Data from the blood parameters are presented in Table 3 according to the stone type. There were no significant differences in the blood parameters among the 6 groups. Also blood analytes were not significantly correlated with PPLA scores.

**Table 3:** Blood and urine data (mean ± standard deviation)

	CaO_*x*_	Apatite	UA	Struvite	MSK	NSA	*p* value
	*n* = 25	*n* = 5	*n* = 5	*n* = 5	*n* = 2	*n* = 4	
*Plasma*							
eGFR (ml/min)	78 ± 16	87 ± 5	62 ± 17	68 ± 26	90 ± 0	76 ± 16	0.1587
Creatinine (μmol/l)	83 ± 21	70 ± 9	105 ± 19	89 ± 50	63 ± 8	77 ± 11	0.2105
Urea (mmol/l)	5.8 ± 1.7	3.8 ± 0.7	6.8 ± 2.4	6.8 ± 3.5	3.8 ± 2.7	6.1 ± 0.6	0.1101
Sodium (mmol/l)	140 ± 2	141 ± 1	141 ± 2	140 ± 4	140 ± 1	142 ± 2	0.3138
Potassium (mmol/l)	4.0 ± 0.3	4.0 ± 0.4	4.1 ± 0.4	3.8 ± 0.2	3.8 ± 0.4	3.9 ± 0.3	0.6912
Ionized calcium (mmol/l)	1.35 ± 0.34	1.35 ± 0.20	1.31 ± 0.08	1.29 ± 0.09	1.25 ± –	1.33 ± 0.09	0.9968
CO_2_ (mmol/l)	26.9 ± 2.1	24.8 ± 3.6	26.4 ± 2.3	26.5 ± 2.1	21.5 ± –	28.3 ± 2.1	0.1308
Phosphate (mmol/l)	0.90 ± 0.19	0.89 ± 0.24	1.00 ± 0.33	1.02 ± 0.17	0.76 ± –	1.11 ± 0.16	0.4245
*Urine*							
Volume (ml)	1686 ± 824	2538 ± 345	1679 ± 552	2158 ± 1136	1348 ± –	2656 ± 921	0.1532
pH	5.9 ± 0.7	7.0 ± 0.4	5.6 ± 0.5	7.2 ± 1.2	6.3 ± 1.1	5.9 ± 0.3	0.0017
Citrate (mmol/24-h)	2.63 ± 2.10	4.48 ± 1.51	2.35 ± 2.37	1.43 ± 1.16	0.40 ± –	3.33 ± 1.47	0.2996
Oxalate (μmol/24-h)	341 ± 326	323 ± 33	296 ± 152	373 ± 136	268 ± –	294 ± 15	0.9967
Calcium (mmol/24-h)	4.36 ± 2.90	7.82 ± 6.65	1.83 ± 0.50	4.73 ± 2.86	4.00 ± –	8.20 ± 2.84	0.0781

Urine pH was measured in fasting spot urine

*CaO*_*x*_ calcium oxalate, *UA* uric acid, *MSK* medullary sponge kidney, *NSA* no stone analysis

### Urine data

Data from 24-h and spot urines are presented in Table 3 according to the stone type. Except for urine pH, there were no significant differences in urinary parameters between groups. Urine pH was significantly lower in uric acid stone formers (5.6 ± 0.2) compared to struvite (7.2 ± 0.2) (*p* = 0.009) and apatite (7.0 ± 0.2) (*p* = 0.03) stone formers; and both struvite and apatite stone formers had significantly higher urine pH than CaO_*x*_ stone formers (5.8 ± 0.1) (*p* = 0.002 and *p* = 0.008, respectively). The patient with primary hyperthyroidism had a very high excretion rate of calcium (17.7 mmol/day) and a high urine pH (Fig. 6).

Rank correlation with all patients in a single group failed to show any significant trends between mean PPLA score and urinary analytes (Table 3). Urinary citrate excretion in females did correlate inversely with total mean PPLA score (*p* < 0.05), but removal of 1 patient from the cohort (who had the highest mean PPLA score, of 5.25) rendered this relationship not significant (*p* = 0.07). Also, there were no significant correlations between the mean scores of the different domains of the PPLA (DP, SP, LC, and RP) and the measured 24-h urinary parameters.

## Discussion

The present series has confirmed that renal stone formers do have distinct papillary changes that may be characterized using the domains of the PPLA scoring system: DP, SP, LC, and RP [14]. Pathogenesis of renal stone formation is apparently diverse. Three pathways of human kidney stone formation have recently been proposed [5, 6]. The first pathway is overgrowth on interstitial apatite plaque (Randall’s plaque), which has been suggested to be the main pathway in idiopathic CaO_*x*_ nephrolithiasis; in the second pathway crystals deposit in the renal tubules as the starting point for renal stone formation; and the third pathway implies free solution crystallization as seen in patients with urinary stasis [6]. Our data clearly show that digital endoscopy of the renal collecting system has the potential to differentiate among these underlying pathways. Although our series included a cohort of stone formers with different types of stone composition/stone disease, it was evident that patients with predominantly DP differed from patients with a high degree of RP, in the sense that patients with DP score > 0 almost all had RP score close to 0 (Fig. 3), indicating different underlying stone pathways. These findings were not reflected in clear differences in urinary parameters. Hence, we were not able to confirm previously published data by Kuo et al. that urine calcium, pH and volume predict coverage of renal papilla by Randall’s plaque [11]. Our data are in line with the study of Linnes et al. [20], who also were not able to show a clear-cut correlation of plaque with urinary factors. In contrast to our population, the series of Kuo et al. [11] consisted of a larger number of hypercalciurics with heavy plaque. Our cases clearly illustrate that hypercalciuria may be associated with both plaques and plugs (Figs. 4 and 6). Thus, from our data it seems evident that patients clinically defined as ‘idiopathic CaO_*x*_ stone formers’ are not necessarily characterized by identical stone-forming pathways. This was also suggested in the paper of Linnes et al. [20]. Efforts to prevent stone formation in idiopathic CaO_*x*_ nephrolithiasis have so far been insufficient [21]. Prevention strategies have almost exclusively been based on final urine studies. Since final urine data according to our findings do not clearly reflect a specific pathogenic pathway, treatment aimed at correcting abnormal urine findings thus may not target the cause of stone formation, explaining why preventive therapy is not always effective. In this perspective, phenotypic characterization of kidney stone formers with the aid of modern endoscopy may hold promise for a more individualized and effective approach to prevention.

As expected, uric acid stone formers in our series had acidic urine [22]. We found uric acid stone formers to have both RP (40%) and DP (60%), confirming previously reported data [16, 20]. Although the nature and pathogenesis of plaques and plugs among uric acid stone formers remain to be defined, these findings combined with the observations in the CaO_*x*_ group may be explained by the fact that CaO_*x*_ and uric acid stone formers often share common systemic characteristics (metabolic syndrome) [23].

In a previous study on biopsy proven MSK, it was found that the most likely mechanism for stone formation in MSK appears to be crystallization due to urinary stasis in dilated inner medullary collecting ducts with subsequent passage of ductal stones into the renal pelvis, where they serve as nuclei for stone formation [19]. Definitely, DP was the most prominent finding in our limited series of MSK patients, thus supporting previous findings. The MSK patients had a very recognizable pattern of papillary malformation during endoscopy. The clinical phenotype of MSK is unclear because patients with other causes of stone formation may be incorrectly labeled as MSK based on radiologic image studies [24]. Therefore, the diagnosis of MSK maybe should be based on endoscopical rather than radiological findings, which potentially may result in more homogeneous metabolic findings in this unique population in the future.

Our apatite stone formers were predominantly characterized by DP and only very limited RP, which was also the main finding in the study of Evan et al. [12]. LC was a characteristic finding in our apatite group, suggestive of advanced papillary injury in this group [14]. Our findings support apatite stone formation to be distinctly different from idiopathic CaO_*x*_ nephrolithiasis and that these patients may be at higher risk for developing chronic kidney disease [25].

The only consistent finding in the struvite group was LC, which may correlate to interstitial inflammation as demonstrated in a recent study [26]. In line with our data, their findings suggest that RP has a limited role for struvite stone formation and an alternate pathogenic mechanism is thus implicated [26].

### Strength and limitations

A strength in our analysis was that stones were classified with both IR and micro-CT, ensuring a high accuracy in characterizing stone composition [6]. A limitation was that stones were sampled retrogradely, meaning that the majority of stones were collected subsequent to laser fragmentation and only a subset of stones (papillary stones and smaller free stones that were extracted in toto) could be considered complete stones. This might have affected classification of patients. Furthermore, the relatively small heterogeneous patient population constitutes a limitation, although we attempted to control for this with a prospective, standardized design. The frequency of compound papillae among the patients in our study likely had an effect on the measures of papillary properties, as there are as yet no published guidelines of what would constitute LC in a compound papilla. Similarly, percentage coverage of pitting or plaque, or numbers of plugs/dilated ducts could be affected by this morphology. For example, a fully compound papilla presumably represents the same quantity of renal function as 2 single papillae [27], but if it had 5 or more plugs/dilated ducts, we scored it a 2 for plugging, which would have increased the average plugging score relative to the same number of plugs/dilated ducts distributed between 2 simple papillae.

## Conclusion

Digital endoscopy revealed that kidney stone formers have different and distinct papillary morphologies that seem to be linked to specific stone-forming pathways. It was evident that patients with predominantly ductal plugging differed from patients with a high degree of Randall’s plaque. Since renal papillary abnormalities may be easily identified during endoscopic surgery, this may in the future prove to be an important method for tailoring prevention strategies in kidney stone patients.
